# Exposure to loneliness cues reduces prosocial behavior: Evidence from N400 and P300

**DOI:** 10.3389/fpsyg.2023.1094652

**Published:** 2023-04-17

**Authors:** Meiling Yin, Eun-Ju Lee

**Affiliations:** ^1^Business School, Sungkyunkwan University, Seoul, Republic of Korea; ^2^Neuro Intelligence Center, Sungkyunkwan University, Seoul, Republic of Korea

**Keywords:** loneliness, prosocial behavior, ERP, frontal N400, posterior P300

## Abstract

Loneliness is a major risk factor for morbidity and mortality. However, the effect of loneliness on subsequent prosocial behavior is not well known. Understanding the neurobiological mechanisms underlying loneliness is necessary to address this research gap. We investigate the mechanism using a modified public goods game (PGG) wherein participants can choose to act for a collective or selfish interest after being exposed to loneliness cues. Both behavioral (Study 1) and event-related potential (ERP) (Study 2) measures were used to explore this relationship. In Study 1 (*N* = 131), we found that participants exhibited decreased prosocial actions under the loneliness priming condition as opposed to the control condition. In Study 2 (*N* = 17), frontal N400 and posterior P300 components were identified under the loneliness priming condition as opposed to the control condition. Increased (decreased) frontal N400 and posterior P300 lead to selfish (prosocial) choices. These results indicate that humans instinctively perceive loneliness as inconsistency with their desired social-relational life, which in turn stimulates coping strategies for self-preservation. This study contributes to our understanding of the neurobiological basis of loneliness associated with prosocial behavior.

## 1. Introduction

Human survival and reproduction require humans to have close relationships with others (DeWall et al., 2011), and positive and lasting relationships represent fundamental human needs (Kothgassner et al., 2017). People invariably prefer living in social groups and performing the necessary actions—even at the expense of minor personal interests—to belong to the group (Hollenbeck and Zinkhan, 2006). Thus, an individual’s social connection results in actions to improve society, such as volunteering their time to help others.

However, perceived social disconnection is associated with loneliness (Wu et al., 2020). Hu et al. (2020) stated loneliness reflects a discrepancy between desired and actual interpersonal relationships, whereas Shevlin et al. (2014) stated that loneliness is a subjective experience of dissatisfaction with one’s social-relational life. We define loneliness as the perception of social isolation and the discrepancy between desired and actual social relationships. The discrepancy between ideals and reality is an experience of loneliness, as people tend to desire positive relationships, not isolation or exclusion (Heu et al., 2019). Previous research has demonstrated that even brief experiences of loneliness threaten the individual’s psychological well-being and physical health (Zadro et al., 2004). Loneliness is a negative emotional experience that increases uncertainty, sadness, and anger and decreases happiness (Cacioppo et al., 2016). Loneliness is closely related to interpersonal hostility, anxiety, and depression (Gopinath et al., 2009; Bedard et al., 2017). One treatment for loneliness is that individuals go out, be with and connect with others (Kellezi et al., 2019; Lapena et al., 2020). In a post-pandemic society affected by social distancing and isolation, we need to reach out to others beginning with prosocial and friendly gestures. Prosocial behavior can be a natural and easy way to combat loneliness and initiate social connections (Miles et al., 2022). Since loneliness is associated with altered social function in specific brain regions and causes severe psychological and mental consequences that cannot be cured naturally (Lam et al., 2021), thus requiring research on the relationship between loneliness and prosocial behavior (Huang et al., 2016; Van de Groep et al., 2020).

Prosocial behavior benefits others more than oneself; therefore, it frequently entails providing resources to others and sacrificing one’s own interests (Debono et al., 2020). Although prosocial behavior has enormous benefits for the collective good, social relationships, and well-being in the long run, maximizing individual interests often make more sense in the short run. In existing studies, the public goods game (PGG) is used as a prosocial task to enact personal and collective interests (Fehr et al., 2002; Filkowski et al., 2016).

PGG originates from behavioral economics, and at its nature is incentives and the problem of free riding (O'Gorman et al., 2009). In this game, subjects secretly choose the number of private tokens to put into a public pot, and then the tokens in this pot are multiplied by a factor, and this “public good” payoff is divided equally among the players, so that non-contributors also keep the tokens (Hasson et al., 2010; Fosgaard and Piovesan, 2015). In the PGG, a common goal is achieved when numerous participants cooperate, and the fruit of cooperation is distributed to all participants, including both prosocial and non-prosocial individuals. Those who do not contribute yet enjoy free-ridden benefits are selfish and a participant’s decision to contribute to the collective interest can be considered a prosocial act to strengthen the public interest (Milinski et al., 2006). Achieving common prosperity and dealing with climate change are examples of public goods that are relevant to the current times (Milinski et al., 2006). Here, climate can be viewed as a public good, and free riding refers to those who enjoy environmental benefits without paying a premium for environmental protection. Therefore, we use a modified PGG, wherein one can choose to act for climate protection or personal benefit.

Previous studies have suggested that loneliness affects individuals’ choice to engage in prosocial behavior (Twenge et al., 2007; Hu et al., 2020). Hence, these two behavioral outcomes are plausible. On the one hand, individuals who experience loneliness behave antisocially or aggressively to avoid threats and reconstruct control (Carter-Sowell et al., 2008; Wang et al., 2012). The human brain has evolved to place individuals into short-term self-preservation mode when they are alone and have no interconnections (Grennan et al., 2021). To avoid threats, they temporarily shut down their emotional systems through over-vigilance toward themselves and emotional insensitivity toward others in potentially threatening situations (Twenge et al., 2001). On the other hand, loneliness can also motivate an individual to increase prosocial attempts to create social bonding and belong to a group (Cacioppo et al., 2016). Loneliness as the emotional state of social isolation increases individuals’ prosocial attempts under certain boundary conditions. Wang et al. (2012) identified that socially excluded participants conformed more to the norm than included participants to minimize others’ negative evaluations.

Therefore, wide research gaps exist regarding whether loneliness increases or reduces prosocial behavior. Notably, loneliness correlates with social behavior; hence, it is of significant value to explore the neurobiological basis of loneliness as it relates to prosocial behavior. This study aimed to explore neural signals to determine how people feel lonely and their effects on prosocial behavior. Therefore, we examined the influence of loneliness on prosocial behaviors using both behavior and electroencephalogram (EEG) studies. Using event-related potential (ERP) analysis technique, we can examine the psychological mechanism that is inherent in loneliness and the activity intensity in specific brain regions.

People want to be liked, included and accepted by other people (Zhu et al., 2018). Considering the fundamental nature of the need for belonging, social isolation precipitates significant discrepancies in the quantity or quality of social contact that individuals desire. The N400 is a negative-going deflection occurring 200–600 ms after stimulus onset, with a slight right hemisphere bias (Kutas and Federmeier, 2011). Studies on the semantic priming effect using ERP have demonstrated that a more negative N400 component can be evoked when the target and prime are semantically incongruent (Draschkow et al., 2018). Zhu et al. (2018) found that larger N400 was induced in exclusionary verbs than in inclusive verbs because the former violates interpersonal self-positivity. Moreover, the N400 can be triggered by social conflicts and violations (Huang et al., 2014). Further, Cacioppo et al. (2016) reported that loneliness is associated with a focus on threatening emotions and that lonely people exhibit an automatic (unconscious) attentional bias toward social threats, such as social rejection. Cacioppo and Hawkley (2009) observed that loneliness is associated with increased activation of the visual cortex when presented with unpleasant social images. The P300 is a positive-going deflection occurring 200–450 ms after stimulus onset (San Martín et al., 2016). This component is associated with visual attention to new stimuli (Yeung and Sanfey, 2004; San Martín et al., 2016), and its amplitude is higher in response to threatening than to neutral faces (Batty and Taylor, 2003; Holmes et al., 2008). Gray et al. (2004) reported that sensitivity to self-relevance cues induces P300. When people experience loneliness, their sensitivity and attention to negative social cues increase (Pickett and Gardner, 2005). Thus, these features of loneliness can affect subsequent social behavior.

Prosocial behavior involves considering another person’s viewpoint with the intention of benefiting them (Batson and Powell, 2003). When the fundamental need for belonging is not fulfilled, individuals adopt a loneliness-perpetuation perspective (Vanhalst et al., 2015). Numerous previous studies have demonstrated the relationship between the neural response to loneliness and vulnerable behavior (Girardi et al., 2009; Serafini et al., 2018; Lam et al., 2021). Research has revealed that owing to loneliness, the prefrontal cortex, which is involved in understanding others’ minds, increases regional gray matter, which induces immature functions in emotion regulation (Kong et al., 2015). Serafini et al. (2020) found abnormal levels of inflammation in specific brain regions are associated with depression and suicidal behavior. Additionally, loneliness decreased the ability to filter less relevant stimuli (Tian et al., 2017), which is associated with hypervigilance (Cacioppo et al., 2014). This attentional bias increases sensitivity to self-relevant information and decreases sensitivity to other-relevant information (Teoh et al., 2020).

In summary, this study aimed to investigate the effect of loneliness exposure on prosocial behavior. Further, we investigate the electrophysiological mechanisms of exposure to loneliness. Our main hypothesis is that social discrepancy and attentional biases toward stimuli that individuals experience after being exposed to loneliness stimuli induce the individual’s frontal N400 and posterior P300. Also, these neural responses focus on self-preservation, which will reduce prosocial behavior.

## 2. Materials and methods

### 2.1. Study 1

#### 2.1.1. Participants

The survey was conducted using Prolific,^1^ an online survey platform for data collection, participants were recruited globally. Our survey’s first page elucidated the study’s purpose and stated that the anonymity of responses provided during the experiment would be ensured. Participants who agreed to the experiment were randomly assigned to one of two conditions (loneliness or control) and saw scenarios related to their respective conditions. Overall, there were one hundred and thirty-one participants with a mean age of 31.9 (SD = 6.7) years. Sixty-six participants (47 women, 19 men) were in the loneliness priming condition, and sixty-five participants (49 women, 16 men) were in the control condition. Based on previous studies the sample size of 25 is adequate for per treatment condition (Maxwell, 2004; Hertzog, 2008; Park et al., 2022). Additionally, G-power 3.1.9 software was used to determine the appropriate sample size for the study. Our study included sufficiently more participants than the number of samples required for power = 0.8 and *α* = 0.05 in the between-subject design (Prajapati et al., 2010).

#### 2.1.2. Stimuli and procedure

The behavioral experiment consisted of two-condition (Loneliness vs. Control) between-subject design. Prior to the experiment, participants’ emotional states of felt loneliness were measured using the University of California, Los Angeles Loneliness scale (UCLA; Russell, 1996), which comprised 10 items (e.g., “I feel alone,” “I feel left out”) on a 7-point scale; moreover, the measurement item’s internal consistency was found to be acceptable (Cronbach’s alpha = 0.86). Higher scores indicated higher loneliness levels.

Our experimental design followed Twenge et al.’s (2007) experimental design and, additionally, presented images corresponding to the conditions. In the control condition, participants read the following message: “You have a lot of relationships, and you have many friends who can help you in difficult times. The images below reflect your situation.” To facilitate the participant’s imagination, we presented group-level images related to social interactions to reflect the current situation. In the loneliness priming condition, participants read the following message: “No one understands you, and no one to talk to. Everyone you love left you. You are always alone. The images below reflect your situation.” To facilitate the participant’s imagination, we presented images of a solitary individual related to social isolation to reflect the current situation. Images were presented using the criteria of the number of people in the image and the closeness of relationships depicted. The images reflect social bonding and loneliness, like the photos used by Silva et al. (2017). The number of images for each condition was fifteen, and each condition was repeated twice.

Loneliness is related to participants’ self-perception of loneliness and is accompanied by negative emotions such as dissatisfaction with social relationships. Therefore, we conducted a manipulation check for the case after the exposures. The loneliness scale was measured again, and the internal consistency of the measurement items was found to be acceptable (Cronbach’s alpha = 0.96). Additionally, we measured bipolar pairs of emotional responses (happy/unhappy, pleased/annoyed, satisfied/unsatisfied, contented/melancholic, hopeful/despairing, and relaxed/bored) on a scale of 1 to 7 (Bradley and Lang, 1994). The internal consistency of the measurement items was found to be acceptable (Cronbach’s alpha = 0.94); wherein higher scores indicate higher levels of negative emotions.

At the end of each condition, participants were asked to choose between eco-friendly and conventional products for five product categories: a new household drain cleaner, a lamp, batteries, bottled water, and shampoo. Following Lee et al.’s (2014) task design, eco-friendly products were described as “good for the environment, but 20% more expensive than conventional products” and non-eco-friendly products were described as “the same price as conventional products.” The prosocial task score was presented as the percentage of respondents choosing eco-friendly products. To increase the experiment’s realness, we informed the participants that the rewards comprised basic money and bonuses, and the higher the eco-friendly selection rate, the smaller the actual bonus they would receive. All participants received $0.5 as a basic amount plus an additional $0.1 for choosing a non-eco-friendly product.

PGG was designed to examine prosocial behavior in groups. In traditional games, participants receive money and decide whether to keep it or donate it to help others; helping others is prosocial. However, if one focuses on personal interests, accepting a free ride without donations would seem preferable. However, such free riding threatens societal welfare. In our study, we replaced donations with eco-friendly consumption to increase participants’ participation by presenting tasks such as the choices they make in their daily lives. Maintaining the Earth’s climate is the biggest “public goods game” played by humans (Milinski et al., 2006). Climate protection is a public good for all of us. Altruists pay more for climate protection and purchase eco-friendly products, but free-riders free-ride on climate protection by purchasing conventional products at relatively low prices.

#### 2.1.3. Behavioral results

Participants reported no difference between the two conditions (loneliness vs. control) in the level of loneliness they felt prior to the experiment (*M*_loneliness_ = 3.10 [SD = 1.17], *M*_control_ = 2.83 [SD = 1.16], *t* [1, 129] = 1.31, *p* = 0.194, Cohen’s *d* = 0.23). However, after the priming treatment, the level of the loneliness felt by the participants significantly differed between the loneliness priming and control conditions (*M*_loneliness_ = 5.38 [SD = 0.73], *M*_control_ = 2.86 [SD = 1.31], *t* [1, 129] = 13.54, *p* < 0.001, Cohen’s *d* = 2.38). Additionally, participants placed in the loneliness priming condition reported a higher level of negative emotion than those in the control condition (*M*_loneliness_ = 5.51 [SD = 1.34], *M*_control_ = 3.10 [SD = 1.63], *t* [1, 129] = 9.22, *p* < 0.001, Cohen’s *d* = 1.62).

According to *t*-test analysis using prosocial behavior as a dependent variable, prosocial behavior was significantly reduced in the loneliness priming condition (*M*_loneliness_ = 0.37 [SD = 0.31], *M*_control_ = 0.49 [SD = 0.34], *t* [1,129] = −2.13, *p* < 0.05, Cohen’s *d* = −0.37). Therefore, the hypothesis that loneliness reduces prosocial behavior is supported.

#### 2.1.4. Discussion

In Study 1, participants experienced more loneliness and negative emotions in the loneliness priming condition and showed reduced prosocial behaviors. Our results are consistent with previous studies showing that social connection enables people to behave sustainably (Abson et al., 2017), and that the experience of loneliness makes it difficult for people to predict future events and empathize with the suffering of others. This indicates that our experimental manipulation is successful. Future research is needed to further investigate the characteristic of loneliness. To better understand individual psychological mechanisms during the experience of loneliness, we investigate electrophysiological mechanisms of loneliness in the context of social behavior.

### 2.2. Study 2

#### 2.2.1. Participants

Twenty right-handed undergraduate and graduate students (9 women, 11 men) with no history of neurological problems were paid to participate in this experiment. Their age ranged between 20 and 29 years (mean = 24.3, SD = 4.1). The procedure was approved by the Institutional Review Board (IRB) of the first author’s university, and written informed consent was obtained from participants before participating in the experiment. Data from three participants were discarded because of the excessive head movements. Finally, data from 17 participants were used for analysis.

In ERP studies, increasing the data’s reliability through repeated measurements is common (Muntean et al., 2021). The sample size was determined based on previous ERP studies (Petrichella et al., 2017; Yun et al., 2022). Additionally, the G-power 3.1.9 software was used to verify the sample size. Our study included sufficiently more participants than the number of samples required for power = 0.8 and *α* = 0.05 in the within-subject design (Prajapati et al., 2010).

#### 2.2.2. Stimuli and procedure

The EEG experiment comprised a one-factor (loneliness priming vs. control) within-subject design. E-prime 3.0 software was used to present the scenario, and the details are similar to Study 1. The EEG experiment for each participant was scheduled in advance and conducted in a soundproof room. Each participant was seated in a comfortable chair, while the experimenter attached EEG electrodes to their scalp.

At the beginning of each condition, there was an instruction to imagine a situation related to the condition and presented images corresponding to the conditions later. Each condition contained 15 images, and two trials were conducted for each condition. The sequences of conditions were counterbalanced across participants. Each image was presented for 3 seconds and a fixation page of a cross sign at the center of the screen was projected for 1 second in between images. After exposure, participants indicated their preference for a conventional product versus an eco-friendly product for five product categories by pressing a corresponding button (1 or 2) on the keypad. The entire EEG experiment took approximately 40 min, after which the subjects were paid $20 for their participation.

#### 2.2.3. EEG recording and analysis

The electroencephalography data were recorded using a 32-channel MR-compatible EEG system (Brain Products GmbH, Germany). Thirty-two Ag/AgCL electrodes (AFz, AF3, AF4, AF7, AF8, F1, F2, F5, F6, FC3, FC4, FT7, FT8, FCz, C1, C2, C5, C6, CPz, CP3, CP4, TP7, TP8, P1, P2, P5, P6, Poz, PO3, PO4, PO7, and PO8) were placed on an elastic cap (actiCap, Brain Products GmbH) according to the standard international 10/20 system. The FCz channel located at the midline frontal-central was selected as the online reference channel (Leuchs, 2019; Yun et al., 2022). All electrode impedances were maintained below 10 Ω during the recording. The EEG signals were continuously sampled at the 500 Hz/channel rate.

**Figure 1:**
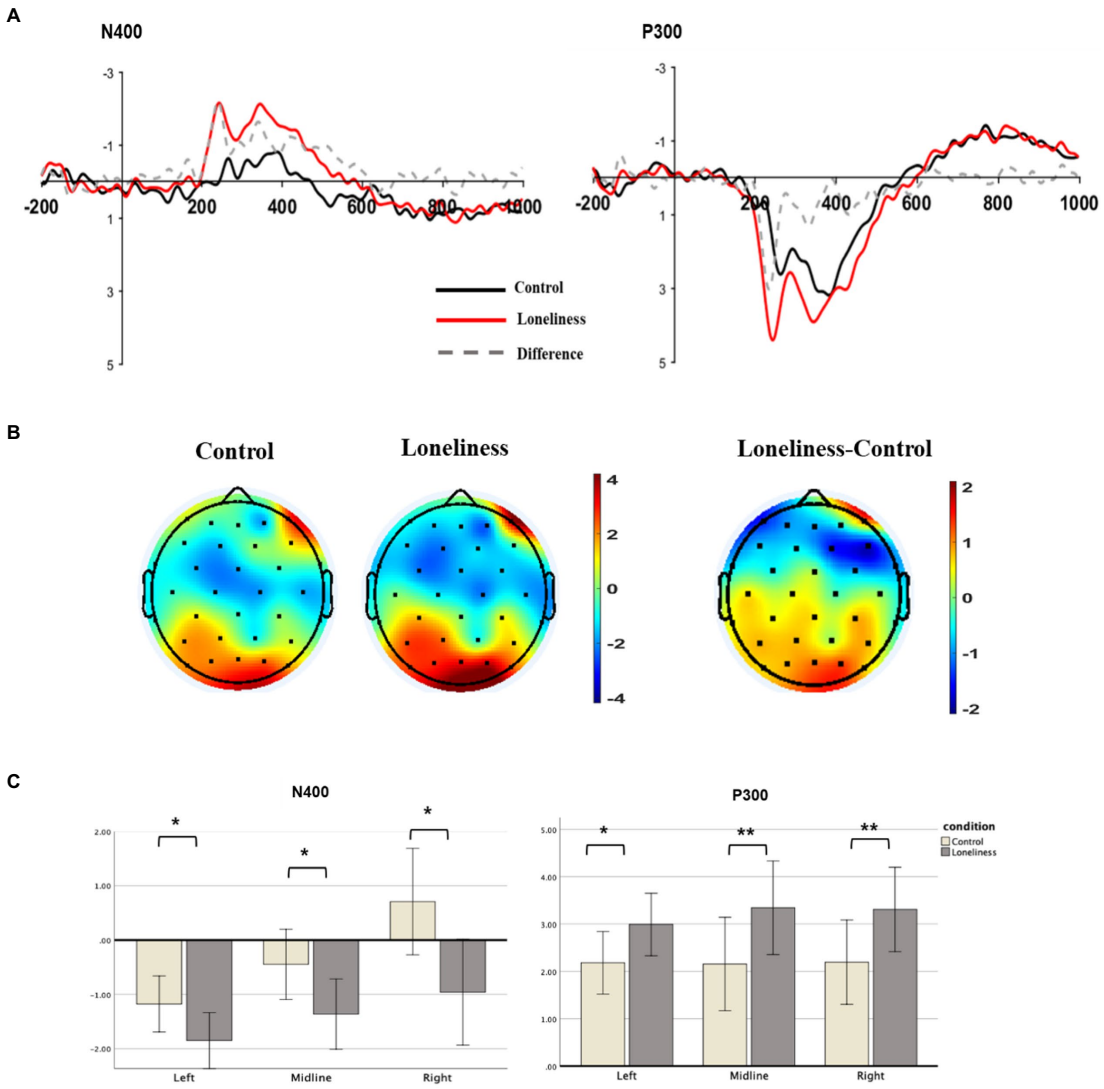
Grand mean ERP results for N400 and P300. **(A)** Grand averaged ERP waveforms on the frontal locations (F1, F2, F5, F6, AFz) and posterior locations (PO3, PO4, PO7, PO8, POz). **(B)** Topographical map for each condition and their difference at the time course from 200 to 500 ms. **(C)** Bar graph depicting mean N400 and P300 amplitudes at the frontal and posterior areas for control vs. loneliness priming conditions. The asterisk indicates a reliable difference between control vs. loneliness priming conditions. Error bars indicate standard error.

Further data processing was performed using EEGLAB and ERPLAB (Lopez-Calderon and Luck, 2014) in MATLAB. All signals were re-referenced to the average of all channels and band-pass filtered with cutoffs at 0.05 and 30 Hz. The BSS-based electro-oculograms (EOG) procedure was applied to correct ocular artifacts (Gómez-Herrero et al., 2006). This method enables the researcher to detect ocular movements and movement-related artifacts without necessarily attaching EOG reference channels. To compute ERPs, continuous EEG was segmented in epochs of 1,000 ms, time-locked to stimulus onset, and included a 200 ms pre-stimulus baseline. EEG voltage amplitudes that exceeded a threshold of ±75 μV during the recording were excluded from the final analysis. According to the grand average ERP waveforms and topographic map (Figure 1), ten electrode sites were selected for statistical analysis as follows: five F channels (F1, F2, F5, F6, AFz) and five PO channels (PO3, PO4, PO7, PO8, and POz). The N400 and P300 components were calculated at mean amplitudes within 200–500 ms and 200–450 ms time windows, respectively. After applying the Greenhouse–Geisser correction, we performed a 2 (condition: control, loneliness) × 3 (laterality: left, midline, right) repeated-measures ANOVA for the N400 and P300.

#### 2.2.4. ERP results

After applying the Greenhouse–Geisser correction, the repeated-measure ANOVA was performed on the mean amplitudes at frontal locations during 200–500 ms. The main effect based on the conditions of N400 was significant (*M*_loneliness_ = −2.78 μV [SD = 1.44], *M*_control_ = −1.40 μV [SD = 1.02], *F* [1, 16] = 7.71, *p* = 0.013, *η*^2^ = 0.33). The significant difference between the two conditions of N400 (200–500 ms) was identified in five channels located in the frontal lobe—namely, F1, F2, F1, F2, and AFz. The main effect of the laterality (left vs. right vs. midline) was also significant (*F* [2, 32] = 16.17, *p* < 0.001, *η*^2^ = 0.34). No significant differences were found for the interaction between condition × laterality (*F* (2, 32) = 2.23, *p* > 0.1, *η*^2^ = 0.06). Therefore, a stronger N400 response was observed in the frontal lobe under the loneliness priming condition (Figure 1).

The main effect based on the conditions of P300 was significant (*M*_loneliness_ = 3.19 μV [SD = 1.58], *M*_control_ = 2.18 μV [SD = 1.26], *F* [1, 16] = 10.73, *p* = 0.005, *η*^2^ = 0.40). The significant difference between the two conditions of P300 (200–450 ms) was identified in five channels located in the posterior lobe—namely, PO3, PO4, PO7, PO8, and POz. There were no significant differences for the main effect of the laterality (*F* [2, 32] = 0.28, *p* > 0.1, *η*^2^ = 0.01) or the interaction between condition × laterality (*F* [2, 32] = 0.32, *p* > 0.1, *η*^2^ = 0.01). Therefore, a stronger ERP amplitude (P300 potential) was observed in the posterior lobe under the loneliness priming condition (Figure 1).

#### 2.2.5. Correlation between ERP and prosocial behavior

We used EEG experiments to measure the difference in prosocial behavior based on two conditions (loneliness vs. control). The paired t-tests with the percentage of prosocial choices revealed a significant difference in prosocial behavior based on the condition (*M*_loneliness_ = 0.34 [SD = 0.21], *M*_control_ = 0.59 [SD = 0.28], *t* [1, 16] = −4.10, *p* < 0.001, Cohen’s *d* = −1.01).

**Figure 2:**
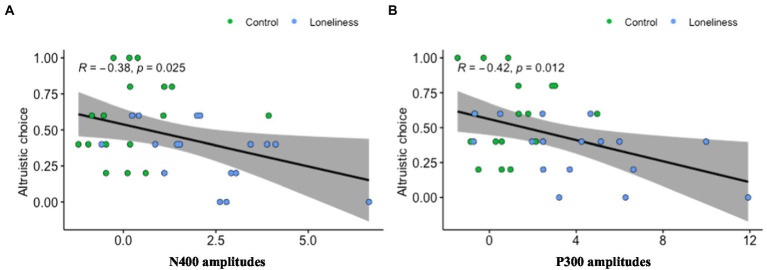
Correlation between N400/P300 and prosocial behavior. **(A)** The relationship between prosocial behavior and the ERP data using two channels from the frontal brain (F1, F2) was highly correlated with prosocial behavior. **(B)** The relationship between prosocial behavior and the ERP data using two channels from the posterior brain (PO3, PO4) was highly correlated with prosocial behavior.

Further, we investigated the relationship between prosocial behavior and the ERP data using four channels—specifically, two channels from the frontal brain (F1, F2) and two from the posterior brain (PO3, PO4), which produced robust neural signals and highly correlated with prosocial behavior. Pearson’s correlation coefficient analysis revealed a significant negative relationship between the frontal N400 signal and the percentage of prosocial choices (*r* = −0.38, *p* = 0.025); see Figure 2. The larger the negative potential in the frontal lobe, the lower the prosocial behavior. Similarly, a significantly negative relationship was found between the posterior P300 and the percentage of prosocial choices (*r* = −0.42, *p* = 0.012); see Figure 2. The larger the positive potential in the posterior lobe, the lower the likelihood that an individual would engage in prosocial behavior.

#### 2.2.6. Discussion

In Study 2, we found that participants elicited greater N400 and P300 components in the loneliness condition than in the control condition. Negative components in the frontal lobe have been previously reported in error detection, social threats, and conflict processing tasks (Aarts and Pourtois, 2012; Huang et al., 2014). Specifically, the frontal N400 has been reported in language and semantic inconsistency tasks (West et al., 2005). N400 has been found to be associated with loneliness because humans instinctively perceive ‘social isolation’ as inconsistent with the naturally desired state of interpersonal connection and unity. Similarly, the posterior positivity peak as P300 has been frequently reported in relation to tasks requiring visual attention (Dai and Feng, 2009). We believe that the N400 and P300 reported in this study are essentially similar to previously reported ERP elements. Our results reveal how the brain functions during human exposure to loneliness and how its neural activity is related to subsequent behavioral choices. Our findings on loneliness are associated with prosocial behavior.

## 3. General discussion

In this study, we investigated individual cognitive processes and subsequent actions related to loneliness. Loneliness needs to be understood because it often responds with depressive symptoms and hostile behavior (Serafini et al., 2018). We found that loneliness reduces prosocial behavior (Study 1 and 2). We found that loneliness is accompanied by a discrepancy between ideals and actual social relationships, resulting in a tendency to act in one’s own interests rather than those of the community, which is represented as frontal N400 and posterior P300 components (Study 2).

Social isolation is not limited to the psychologically vulnerable but is a prevalent phenomenon in modern society. However, the social sciences, except in the field of personality disorders, have overlooked this problem. As the human brain is sensitive to loneliness cues, the N400 and P300 components are useful biomarkers for emotional processing associated with loneliness. Loneliness also affects subsequent behavior. The experience of loneliness results in a discrepancy between one’s ideals and actual social relationships, which makes one tend to behave in their own interests rather than for the community’s benefit. Our investigation of the correlation between loneliness-related neural activity and subsequent decision-making supports the notion of a relationship between behavioral responses and neural activity.

Our results are consistent with those of other studies on loneliness in three ways. First, we found that loneliness was caused by an emotional discrepancy between ideal and actual social relationships. The emergence and maintenance of loneliness are associated with the use of emotion-focused coping, which may include emotional suppression, withdrawal, passive resignation, or avoidance, rather than problem-solving and cognitive reconstruction (Huang et al., 2016). Staebler et al. (2011) observed that the lack of meaningful relationships, which is increasing in modern society, increases individuals’ emptiness and rejection. Using the ERP waveforms, we found that negative potential (N400) peaks were activated in the frontal region when participants experienced loneliness. In short, participants’ loneliness induces a discrepancy between the ideal and actual relationship, represented as an N400 peak in the frontal region.

Second, another component identified after stimulus presentation was the P300 components for loneliness conditions. At the posterior location, the loneliness priming condition elicited a larger positive potential peak than the control condition. Positive activity in the posterior lobe is involved in attention and visual perception (Woodman, 2010), which is consistent with the notion that the lonelier one feels, the more attention one pays to negative stimuli or threats (Cacioppo et al., 2016). Neuroplasticity may make individuals more sensitive to negative life events (Belsky et al., 2007). Painful experiences, such as loneliness, require greater attention because they are related to survival. This constant attention and vigilance that results from loneliness tend to focus on the self. In short, the participants’ loneliness induced attentional bias, which was represented as a P300 peak in the posterior brain region.

Third, we used the PGG, which found that participants exhibited lower prosocial behavior in the loneliness priming condition. Individuals focus on self-benefits when they are socially isolated (Yu and Han, 2021). This is consistent with previous research that prosocial behavior decreases because loneliness is a painful experience that people want to avoid (Huang et al., 2016). A sense of belonging connects individuals and groups and induces prosocial behaviors, as the emotionally connected approach to sustainability recognizes the intrinsic value of the natural world and seeks to serve community interests. By contrast, loneliness impairs prosocial behavior.

This study has some limitations that provide suggestions for future research. First, we investigated cognitive processes related to loneliness through laboratory manipulations. To obtain more general results, it is necessary to measure it integrated with real-life experience of loneliness. Second, studies have suggested that loneliness is associated with impaired social functioning (Jobe and White, 2007; Lam et al., 2021). Future studies can use fMRI to investigate a wider range of brain areas and functions, such as the deep brain limbic system or the default mode network. Third, the negative effect of loneliness on prosocial behavior can lead to different outcomes when making behavioral choices in public or interacting with others (Wang et al., 2012; Huang et al., 2016). Further studies can add other boundary conditions to compare results. Fourth, culture interacts with how loneliness is dealt with (Van Staden and Coetzee, 2010). Because the Western culture emphasizes the independent self, and the East Asian culture emphasizes interconnectedness with others, the degree of loneliness people feel varies from collective to individualistic cultures (Yum, 2003). Future research should include samples from different cultures.

Despite the above mentioned limitations, our study also provides further insights into the effect of loneliness not only on cognitive processes but also on prosocial behaviors. The whole brain markers that repeatedly appear in ERP results—specifically, the frontal N400 and posterior P300—are a reliable way to detect loneliness. As the interaction between computers and humans has increased recently, we suggest that these neural indicators can provide a service that can generate and recharge positive energy by detecting human loneliness. In addition, a sensitive response to loneliness cues further inhibits an individual’s prosocial behavior and leads to a vicious cycle of relationships. Since loneliness is related to an altered immune system and psychosocial impairment, individuals have limitations in self-healing. It suggests that social support such as a community and healthcare service that can connect with others is needed to induce altruistic behavior among lonely individuals.

## 4. Conclusion

We investigate the psychological and neural mechanisms of loneliness, adding understanding to prosocial behavior. The loneliness priming condition elicited larger frontal N400 and posterior P300 amplitudes than the control condition. In addition, these neural markers have been found to be associated with subsequent prosocial behavior. The findings help us better understand why loneliness reduces prosocial behavior and what measures can be taken to improve prosocial behavior.

## Data availability statement

The original contributions presented in the study are included in the article/supplementary material, further inquiries can be directed to the corresponding author.

## Ethics statement

The studies involving human participants were reviewed and approved by the Institutional Review Board (IRB) of Sungkyunkwan University (2021-12-018). The patients/participants provided their written informed consent to participate in this study.

## Author contributions

MY: collected and analyzed the data and wrote the manuscript. E-JL: project administration and designed the experiments. All authors contributed to the article and approved the submitted version.

## Funding

This research is funded by Korea National Research Foundation (NRF) (2021R1A2B5B01001391) awarded to E-JL.

## Conflict of interest

The authors declare that the research was conducted in the absence of any commercial or financial relationships that could be construed as a potential conflict of interest.

## Publisher’s note

All claims expressed in this article are solely those of the authors and do not necessarily represent those of their affiliated organizations, or those of the publisher, the editors and the reviewers. Any product that may be evaluated in this article, or claim that may be made by its manufacturer, is not guaranteed or endorsed by the publisher.

## Footnotes

^1^ https://www.prolific.co
